# Spouses, social networks and other upstream determinants of type 2 diabetes mellitus

**DOI:** 10.1007/s00125-018-4607-1

**Published:** 2018-04-13

**Authors:** Joreintje D. Mackenbach, Nicole R. den Braver, Joline W. J. Beulens

**Affiliations:** Department of Epidemiology and Biostatistics, Amsterdam Public Health Research Institute, VU University Medical Center, De Boelelaan 1089a, 1081HV Amsterdam, the Netherlands

**Keywords:** Diabetes, Environmental drivers, Lifestyle behaviours, Obesity, Prevention, Social networks, Systems science, Upstream determinants

## Abstract

Diabetes risk factors outside the individual are receiving increasing attention. In this issue of *Diabetologia*, Nielsen et al (DOI: 10.1007/s00125-018-4587-1) demonstrate that an individual’s obesity level is associated with incident type 2 diabetes in their spouse. This is in line with studies providing evidence for spousal and peer similarities in lifestyle behaviours and obesity. Non-random mating and convergence over time are two explanations for this phenomenon, but shared exposure to more upstream drivers of diabetes may also play a role. From a systems-science perspective, these mechanisms are likely to occur simultaneously and interactively as part of a complex system. In this commentary, we provide an overview of the wider system-level factors that contribute to type 2 diabetes.

## Introduction

Despite major investment in research and treatment options, type 2 diabetes mellitus remains a pressing public health issue that is approaching epidemic proportions globally [1, 2]. Excess weight is an established risk factor for type 2 diabetes and the global epidemic of obesity largely explains the major increase in its prevalence in recent decades. Behavioural risk factors for type 2 diabetes include a poor diet, physical inactivity, stress and poor sleep quality [3]. Type 2 diabetes [4], obesity [5] and behavioural risk factors [6] are socioeconomically patterned, with individuals at lowest socioeconomic position (SEP) being at the highest risk.

## Upstream determinants of type 2 diabetes

There is increasing recognition that the conditions in which individuals are born, grow up, live, work and age are important for understanding the aetiology of type 2 diabetes. There is a growing evidence base for such upstream determinants of health [7]; for example, adults living in rural areas have a lower risk of type 2 diabetes. Also, more walkable areas and more greenspace are associated with a lower risk of type 2 diabetes, probably owing to a higher level of physical activity in these areas [8, 9]. Moreover, availability, accessibility and affordability of food is associated with dietary intake and may, therefore, be associated with type 2 diabetes [10], whilst noise pollution may increase the risk of type 2 diabetes via disrupted sleep patterns [11]. Although individual-level factors, such as genetic, biological and psychological factors, remain of importance, they are unlikely to fully explain the enormous increase in type 2 diabetes incidence over the past decades. Hence, the research focus is shifting to more upstream determinants of health, which may be of importance for the early identification of individuals at high risk of type 2 diabetes and the development of subsequent initiatives to intervene in high-risk populations. In this issue of *Diabetologia*, Nielsen et al [12] contribute to this field of research by investigating the influence of spousal diabetes status and cardiometabolic risk factors for an individual’s diabetes risk.

## Spousal diabetes concordance

The study by Nielsen and colleagues [12], using data from 3649 men and 3478 women included in the English Longitudinal Study of Ageing (ELSA), demonstrated that obesity levels in one spouse were associated with incident type 2 diabetes in the other spouse. Interestingly, having an obese spouse increased the risk of type 2 diabetes in men over and above the effect of their own obesity level, while this was not the case for women. In addition, having a spouse with diabetes was associated with an increased risk of type 2 diabetes in women (incidence rate ratio [IRR] 1.40 [95% CI 0.95, 2.08]) but not in men (IRR 1.02 [95% CI 0.64, 1.65]) [12]. This association in women was not statistically significant but, given the low number of cases of diabetes in this study and the relatively large effect size, this may be regarded as relevant for public health. In general, the nationally representative sample, the long follow-up period and thorough analyses provide confidence in the findings. The implications of the results for clinical practice may, however, be limited since high-risk couples may be concordant in their non-attendance for screening; this should be subject to future investigations.

The results of this study are in line with a previous meta-analysis that demonstrated evidence for spousal diabetes concordance [13] and is in line with studies providing evidence for spousal similarities in lifestyle behaviours and obesity [14, 15]. As Nielsen et al state [12], two commonly used explanations for spousal similarities in behaviour and health are non-random mating and convergence over time [16]: individuals are more likely to select a partner with similar phenotypes and preferences, and over the course of a relationship, spouses converse in their behaviours because of social contagion.

## Impact of social networks on health

These effects outlined above may not be limited to spouses; Christakis and Fowler [15] showed that pairs of friends and siblings of the same sex appeared to have more influence on the weight gain of each other, than pairs of friends and siblings of the opposite sex. The importance of social networks (e.g., social structures composed of interdependent individuals, such as spouses, relatives, colleagues, neighbours and friends) for health has been recognised for decades [17]. Social contacts may shape norms about the acceptability of being overweight or preferences for an active lifestyle or may provide support for behaviour change. Not surprisingly, social influences are an important element of the behaviour change technique taxonomy of Michie et al [18], which is used in many type 2 diabetes prevention strategies. In addition, both risk factors and protective factors may spread through social networks. For example, our recent study in European adults showed that individuals living in neighbourhoods with higher levels of social cohesion and stronger social networks were less likely to be obese than individuals living in neighbourhoods with lower levels of social cohesion and weaker social networks [19].

Following the reasoning above, health behaviours and chronic conditions may not just ‘spread’ via spouses, friends and siblings, but even across entire families, neighbourhoods or cities. If Nielsen et al had had data on cardiometabolic risk factors of other family members, friends or neighbours, they may have found that, not only spousal factors, but wider social environmental factors were associated with risk of developing type 2 diabetes. In turn, these similarities in type 2 diabetes risk across a social network may be explained by lifestyle behaviours, socioeconomic conditions across the lifespan, or exposure to food and physical activity environments. Indeed, a third explanation for behavioural and health similarities between connected individuals is shared exposure to common environmental factors.

## Shared environmental factors and type 2 diabetes

Although Nielsen et al adjusted for SEP, all the relevant socioeconomic variation in type 2 diabetes risk may not have been captured. They used the highest reported employment rate at the couple level to indicate SEP, while socioeconomic condition across the life span, including childhood SEP and parental SEP, and area deprivation, may also explain spousal similarities [20, 21]. For example, a Swedish study on the effects of neighbourhood deprivation showed that refugees assigned to high deprivation areas had increased risk of type 2 diabetes, regardless of individual SEP, with neighbourhood effects growing over time [22].

Unfortunately, Nielsen and colleagues did not have data available on other shared environmental factors and, thus, the authors could not investigate whether such factors may explain spousal similarities and differences in type 2 diabetes risk. They did, however, touch upon the role of the food environment for spousal concordance in type 2 diabetes. They found that a wife’s obesity status was a stronger risk factor for incident type 2 diabetes in her husband than vice versa; they speculate that this may be explained by the fact that women are more likely to be responsible for planning, preparing and shopping for food. Indeed, in couples with a more traditional division of roles, a woman’s unhealthy dietary practices may influence both her own and her husband’s risk of type 2 diabetes, while a man’s unhealthy dietary practices (likely originating from the out-of-home food environment) may not influence his wife’s risk of type 2 diabetes. This is, however, discordant with the finding that triacylglycerol levels in men (which are influenced by diet [23]) can impact upon type 2 diabetes risk in the wife [12]. Before any conclusive statements can be made about spousal or wider social network influences on type 2 diabetes, the effects of common exposure to shared environments should be explored. Follow-up studies could investigate whether lifestyle behaviours, socioeconomic conditions, wider social influences and exposure to food and physical activity environments could explain similarities in type 2 diabetes risk.

## Clinical relevance

**Fig. 1:**
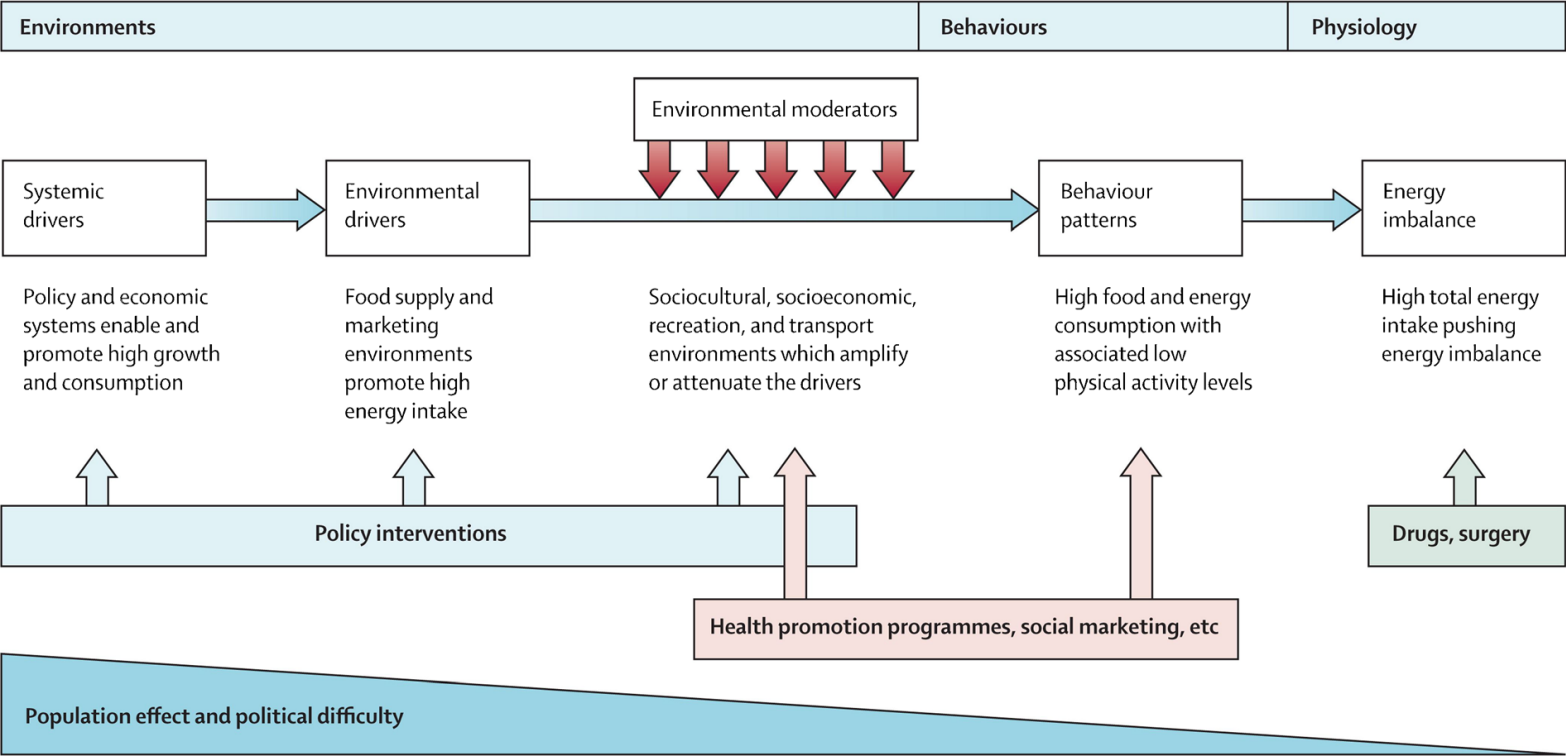
A framework to categorise obesity determinants and solutions. The more distal drivers are to the left and the environmental moderators that have an attenuating or accentuating effect are shown, along with some examples. The usual interventions for environmental change are policy based, whereas health promotion programmes can affect environments and behaviours. Drugs and surgery operate at the physiological level. The framework shows that the more upstream interventions that target the systemic drivers might have larger effects, but their political implementation is more difficult than health promotion programmes and medical services. Although this figure relates to obesity, it is likely that the environmental and systemic drivers shown are also likely to influence type 2 diabetes risk. Reprinted from The Lancet [24], with permission from Elsevier

While the short-term clinical relevance of taking into account such upstream factors in the early detection of type 2 diabetes may be limited, it may still be important to take a step back to see the larger picture. Trying to identify individuals at risk of type 2 diabetes by looking at their ‘nearest neighbour’ (e.g., spouse, sibling or friend) may be regarded as fighting a running battle, given that there are more distal drivers that cause these spouses, siblings and friends to develop type 2 diabetes in the first place. As an example, Fig. 1 displays a framework for obesity as proposed by Swinburn et al [24]. Environmental factors may be viewed as moderators that have an attenuating effect on lifestyle interventions, as such being of relevance to clinicians. Indeed, trying to adhere to dietary recommendations in an obesogenic environment may feel like swimming against the stream: individuals may be able to cope for a while, but then they get tired and give in. Focusing on the environmental and systemic drivers of type 2 diabetes is, therefore, likely to generate a larger preventative population effect, but is politically more difficult. Public, political and media discourse around obesity and diabetes has been dominated by a persistent skew towards individual-level choices as the primary determinant, and this is then reflected in policies and interventions that focus on individual-level behaviour change [25]. These downstream endeavours should not be regarded as negative, as diabetes treatment saves lives and secondary prevention helps to prevent people from developing complications. However, healthcare professionals also have a role to play in raising awareness about primary prevention and could be a major part of the movement towards looking upstream [26].

## Type 2 diabetes as the result of a complex system

It is important to realise that type 2 diabetes (like obesity) is likely to be the result of a complex, adaptive system [27]. There are multiple factors that exert an influence on the development and progression of type 2 diabetes and these factors are likely to interact in a dynamic way. Complex systems are defined by several properties, such as emergence, feedback and adaptation [28]. Emergence refers to the development of an outcome (e.g., type 2 diabetes) that cannot be explained sufficiently by the individual elements in a system, because it is the result of more than the sum of parts. Feedback loops describe the situation in which a change in the system leads to further change; for example, a fast food ban around schools leads to reduced social acceptability of the consumption of fast foods, which leads to a reduced demand for fast food, resulting in reduced supply of fast food. Adaptation refers to adjustments in behaviour in response to changes in the system, for example, a change in the formulation of sugar-sweetened beverages (SSB) in response to an announced SSB-tax. If we can agree that type 2 diabetes is the result of a complex set of interacting factors from within and outside the medical sector, we can see that the problem cannot necessarily be solved with simple, short-term and isolated initiatives. It will likely take actions in multiple areas of the system to bring about a sustainable shift in type 2 diabetes. This encompasses actions that move beyond the direct effects on individuals and focus on reshaping the system itself [28]. A biomedical approach will remain important for type 2 diabetes but, alone, it is unlikely to result in a significant decrease in the prevalence of type 2 diabetes. Hence, healthcare professionals should move beyond a static, clinical view and look at other factors in the patient’s life that may affect disease trajectories, such as spousal risk factors, taking into account that these factors are dynamic, and may interact with and impact on each other over time [29].

## Conclusions

In conclusion, Nielsen et al made an important contribution to the field by explaining the relevance of taking factors external to the individual into account when assessing risk of type 2 diabetes. Indeed, early detection of diabetes risk and subsequent interventions may be improved by using a couple-based, rather than an individual-based, approach. Moreover, healthcare professionals, researchers and policy makers should take into account the wider systemic drivers of the type 2 diabetes epidemic and realise that the effect of downstream interventions may be attenuated by upstream drivers. This implies that a systems response may be necessary to bring about the desired reduction in type 2 diabetes risk. To enable further research into this, broader data collection is required, not only on the influence of spouses, friends and siblings, but also neighbours, other family members and employers, the recreation, transport and food environment, and policy and economic systems.

## Abbreviations

IRR: Incidence rate ratio
SEP: Socioeconomic position
SSB: Sugar-sweetened beverages
